# Pain in Hemophilia: Unexplored Role of Oxidative Stress

**DOI:** 10.3390/antiox11061113

**Published:** 2022-06-03

**Authors:** Raghda Fouda, Donovan A. Argueta, Kalpna Gupta

**Affiliations:** 1 Division of Hematology/Oncology, Department of Medicine, University of California, Irvine, CA 92697, USA; foudar@hs.uci.edu (R.F.); daarguet@hs.uci.edu (D.A.A.); 2 VA Medical Center, Southern California Institute for Research and Education, Long Beach, CA 90822, USA; 3 Division of Hematology, Oncology, and Transplantation, Department of Medicine, University of Minnesota, Minneapolis, MN 55455, USA

**Keywords:** hemophilia, reactive oxygen species, hemarthrosis, antioxidants

## Abstract

Hemophilia is the most common X-linked bleeding diathesis caused by the genetic deficiency of coagulation factors VIII or IX. Despite treatment advances and improvements in clinical management to prevent bleeding, management of acute and chronic pain remains to be established. Repeated bleeding of the joints leads to arthropathy, causing pain in hemophilia. However, mechanisms underlying the pathogenesis of pain in hemophilia remain underexamined. Herein, we describe the novel perspectives on the role for oxidative stress in the periphery and the central nervous system that may contribute to pain in hemophilia. Specifically, we cross examine preclinical and clinical studies that address the contribution of oxidative stress in hemophilia and related diseases that affect synovial tissue to induce acute and potentially chronic pain. This understanding would help provide potential treatable targets using antioxidants to ameliorate pain in hemophilia.

## 1. Introduction

Hemophilia is the most common X-linked chronic bleeding diathesis, caused by the deficiency of coagulation factor VIII (FVIII) or factor IX (FIX) in hemophilia A and hemophilia B, respectively [1]. Despite being a genetic disorder, almost one-third of patients with hemophilia (PWH) do not have a family history of the disease [2]. According to coagulation factor activity level, severity is classified as mild, moderate, or severe. The frequency of bleeding and severity are usually correlated; severe deficiency (plasma FVIII or FIX < 1 U/dL) manifests as prolonged and excessive spontaneous bleeding or secondarily to trauma [3,4,5]. Hemophilia is associated with several clinical manifestations due to bleeding-related long-term complications of which hemarthrosis is the most common and severe [6,7,8]. The early onset and recurrent bleeding result in significant structural joint deformities triggered by the release and accumulation of hemoglobin, iron, and oxygen free radicals in the joint. However, the sequence of events and the molecular mechanisms resulting in joint deterioration are incompletely understood. The morbid effects of hemarthrosis lead to joint damage, physical deformity, chronic pain, and negative effects on health-related quality of life (HRQoL) [9,10].

Hemophilia treatment is mainly replacement therapy using clotting factor concentrates, such as prevention or intervention for acute bleeding. Factor VIII replacement and other therapies reduce the severity and incidence of bleeding, but pain may persist [11]. Pain affects physical and mental health and impairs HRQoL [12,13,14,15]. The lack of evidence-based guidelines for hemophilia-specific pain is recognized globally [12,16,17,18]. Pain is one of the compelling concerns associated with joint damage from the patient’s perspective. Improvements in hemophilia treatment call for a better understanding of the pathobiological mechanisms that lead to disabling arthropathy and associated chronic pain. This review outlines our current knowledge of the pathobiology of hemophilic arthropathy, highlighting the role of oxidative stress as a leading contributor to pain.

## 2. Pain in Hemophilia

Pain is a debilitating consequence of hemophilia. Pain can start early in infancy and continue throughout life with limited treatment options [19,20,21]. The most commonly affected joints are the knees (45%), followed by the elbows (30%), ankles (15%), shoulders (3%), and wrists (2%) [22]. Approximately 70% of patients with severe hemophilia suffer from pain daily, which can be recurrent, acute, and/or chronic [19,23]. Acute pain occurs during joint bleeds, and their repeated recurrence causes bone/cartilage damage leading to arthropathy resulting in chronic pain [3,11,15,24]. Chronic pain is complex and is associated with neurobiological, psychological, and social changes that may maintain the pain [11].

Clinical characteristics of hemophilia pain include ‘throbbing,’ ‘sharp,’ ‘tender,’ ‘aching,’ and ‘nagging’ [23,24]. Female carriers of hemophilia have reduced joint range of motion compared to non-carrier subjects [25]. Joint pain has a negative impact even on simple activities of daily living such as walking. Changes in gait patterns, including walking speed, load bearing asymmetry, swing phase, and stance phase have been observed in patients with severe and moderate hemophilia when compared to healthy subjects [26]. 

In addition to physical impairment (pain, hemophilic arthropathy, organ dysfunction), bleeding episodes in hemophilia often result in mental impairments leading to restriction in activities including school and social participation that affect education and QoL [27]. Joint pain in PWH is associated with reported dependency on pain-relieving drugs and depressive episodes [16]. In children, evidence exists that musculoskeletal dysfunction and associated pain affect the HRQoL, performance, and regular activities of daily living. This can lead to avoidance of many social and physical activities, including sports. Often, there is a fear of pain due to physical activity. For example, a study on children aged 4–12 years found that although motor performance and disability did not differ significantly between children with hemophilia and their healthy peers, most patients perceived that presence of pain had an impact on their performance [28]. In addition, children with hemophilia who experience more bleeding episodes are likely to have poorer academic performance than those with fewer bleeding episodes [29].

The basis of chronic pain caused by recurrent joint bleedings, whether nociceptive or neuropathic, has not been investigated. To address this, the painDETECT-questionnaire (pDq) was used on PWH and control subjects. The pDq identifies neuropathic components in a person’s pain profile. Interestingly, this study showed a positive neuropathic pain component in nine PWH but not in controls and concluded that there is likely a potential risk to misunderstand underlying pain mechanisms in PWH [30].

## 3. Hemarthropathy and Oxidative Stress, Two Faces of the Same Coin

Articular cartilage is designed to bear and distribute the mechanical loads across the joints [31]. The majority of the articular cartilage’s viscoelastic and smooth structure is maintained by chondrocytes, which regulate the production of extracellular cartilage matrix (e.g., type II collagen and aggrecan) and maintain tissue homeostasis [32]. The synovial membrane is a thin specialized soft connective tissue membrane that lines the inner surface of synovial joint capsules. Unlike the avascular articular cartilage, the synovium is well innervated and vascularized, playing an essential role in transporting nutrients, debris and waste removal, immune modulation, and inflammation in the joint [33]. These specialized functions are achieved through specific cell types in the synovial membrane; macrophage-like synovial cells (type A) maintain the synovial fluid by removing wear and tear debris, and fibroblast-like synoviocytes (FLS, type B) are responsible for synthesizing and secreting major extracellular matrix proteins in synovial fluid, such as hyaluronic acid and lubricin [34].

**Figure 1:**
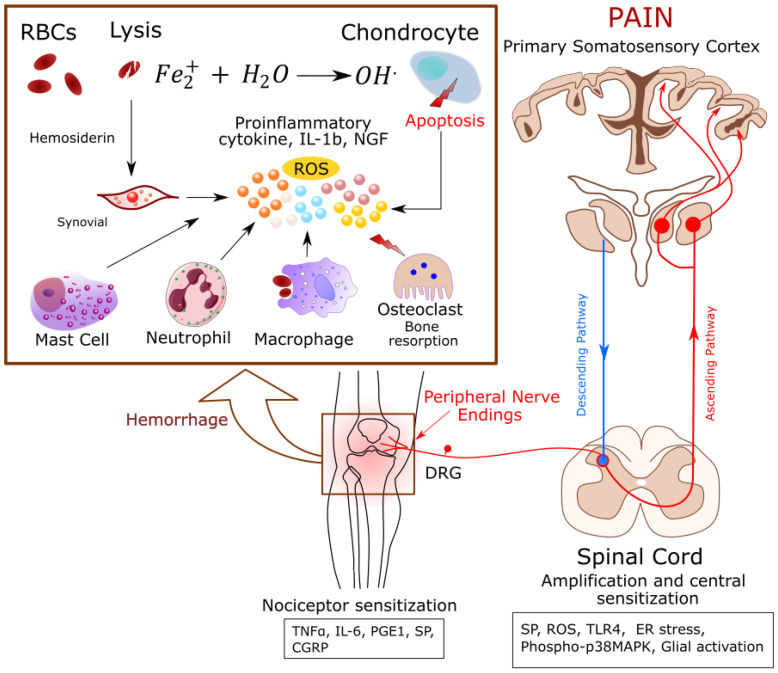
Proposed mechanism(s) of pain in Hemophilia: Joint bleeding releases cell free heme and proinflammatory cytokines leading to synovitis. Red cell derived iron (Fe^2+^) causes oxidative stress and chondrocyte apoptosis. Inflammation activates Receptor Activator of NF-κB Ligand (RANK-L)-RANK-Osteoprotegin (OPG)-pathway, resulting in bone resorption by osteoclasts. This microenvironment of inflammation, cell damage, and oxidative stress activates nociceptors on the nerve fibers, which release neuropeptides and transmit the action potentials to the central nervous system (CNS) leading to the perception of pain. Abbreviations: CGRP: Calcitonin gene-related peptide, DRG: Dorsal root ganglion, ER stress: Endoplasmic reticulum stress, NGF: Nerve growth factor, PGE1: Prostaglandin E1, RBCs: Red blood cells, ROS: Reactive oxygen species, SP: Substance P, TLR4, Toll like receptor 4, TNF-α: Tumor necrosis factor.

The underlying pathogenesis of hemophilic arthropathy is not fully explored. However, evidence shows that recurrent joint bleeding creates a cascade of intra-articular inflammation, angiogenesis, and joint destruction [35]. The development of hemophilic arthritis involves three main stages: acute hemarthrosis, chronic synovitis, and degenerative arthritis [36]. The intra-articular blood effusion favors iron accumulation, released from hemoglobin, and hemosiderin deposition, both of which are believed to be pivotal for the early phase of hemophilic arthropathy, inducing cytokines and pro-angiogenic factors to progressive synovial proliferative growth and ultimately resulting in articular cartilage destruction [37,38,39]. Thus, there is a growing interest in the destructive role of reactive oxygen metabolites as an early pathogenic step resulting in chondrocyte apoptosis, with synovial inflammatory changes being secondary or parallel to the process of cartilage damage [35] (Figure 1).

### 3.1. Cell-Free Heme

Following a joint bleed, the synovium is overloaded with clearing the products of hemolysis. Iron, an essential constituent of hemoglobin found in erythrocytes, is a crucial breakdown product and is thought to play a significant part in inflaming the synovium [40]. The contribution of iron to the hemophilic arthropathy is multifactorial. The iron-rich breakdown product hemosiderin induces the expression of several pro-inflammatory cytokines such as interleukin (IL)-1α, IL-6, and tumor necrosis factor-alpha (TNF)-α [35,41]. Moreover, it appears that iron is involved in the initiation of synovial pannus growth by dysregulating the expression of critical genes such as *c-myc* and *mdm2*, which are responsible for synoviocyte proliferation [42,43,44]. Thus, the synovium becomes increasingly vascular and hypertrophic, and inflammatory cells are recruited to the area in greater numbers in hemophilia [40]. The activated synovium affects cartilage by producing cartilage-destructive pro-inflammatory cytokines and matrix-degrading proteinases [45], resulting in further hemarthrosis and creating a vicious cycle of bleeding and inflammation [46]. Von Drygalski et al. showed that iron deposition can occur in cartilage (not only in synovium) and established the relationship between cartilage hemosiderin in hemophilic joints and joint deterioration [47].

Additionally, iron contributes to direct cartilage damage induced by oxidative stress [48,49]. Iron plays important roles in normal cellular processes including oxygen storage and transport, and energy metabolism. Delicate regulation of iron homeostasis is required for maintaining normal cellular function, while excessive iron would damage cells by increasing the production of reactive oxygen species (ROS). Oxidants, such as hydrogen peroxide (H_2_O_2_), are produced by activated mononuclear cells and chondrocytes [40]. In the presence of erythrocyte-derived iron, H_2_O_2_ generates highly toxic hydroxyl radicals through the Fenton reaction, resulting in the peroxidation of membrane lipids, mitochondrial dysfunction, cellular protein and nucleic acid damage, and ultimately ferroptosis [50], leading to permanent cartilage damage [48,51]. Recent findings indicate that perturbation in iron homeostasis is implicated in the progression of many diseases such as osteoarthritis (OA), osteoporosis, Parkinson’s disease, and Alzheimer’s disease [52]. The association between iron overload and OA pathogenesis is broadly appreciated regarding its effect on ROS production and oxidative stress. OA is characterized by synovial tissue inflammation and cartilage degeneration [53]. Oxidative stress and mitochondrial dysfunction have been demonstrated to be an important contributor to iron overload-induced cartilage degeneration [54,55,56]. Jing et al. further confirmed these observations by showing that iron chelator or antioxidant drugs could inhibit iron overload-induced OA-related catabolic markers and mitochondrial dysfunction [53]. 

Interestingly, a closely related pathogenesis has been observed in sickle cell disease (SCD)-associated OA, with joint space narrowing, synovial inflammation and reduced periarticular bone mass [57]. Retrospective clinical studies indicate that iron overload may have an adverse effect on osteochondral homeostasis in SCD patients [58], as approximately 70% of patients suffering from SCD and exhibiting high serum iron levels had relatively low bone mass. In agreement with these findings, humanized sickle mice showed significant stance instability, perhaps due to pressure pain; reduced walking speed; increased stance duration, with hesitancy perhaps due to anticipation of pain; and reduced diagonal swing, which may indicate poor coordination [59]. These findings further support the contribution of free iron in osteopathic changes leading to altered gait in hemophilia.

### 3.2. Bone Destruction

Repeated bleeding episodes in hemophilia leave the synovial membrane hypertrophied and villous with intense neovascularization. Over time, chronic synovitis is established with pannus, thinning of the cartilage, bony changes, and, eventually, marked joint dysfunction (i.e., reduced range of movement, stiff or unstable joints, swelling, and pain with use) [36]. It is noteworthy that osteoporosis has been frequently reported among PWH and correlated with the severity of arthropathy, which might reflect an imbalance between bone resorption and bone formation [60,61]. A possible mechanism for the bone resorption might be the reduction of thrombin, which is known to mediate the proliferation of osteoblasts via protease-activated receptor-1 (PAR-1) mediated proliferation of osteoblasts [62], shifting the balance towards osteoclastic activity.

Mechanisms underlying bone turnover are regulated strictly with the molecular triad of osteoprotegerin (OPG), the receptor activator of nuclear factor κB (RANK), and the RANK ligand (RANKL) [63,64]. The osteoclast precursors express the receptor RANK on their surface; following binding of RANK to its ligand RANKL, osteoclastogenesis is induced and potentially enhanced by cytokines (e.g., TNF-α, IL-1, and IL-17) that promote both inflammation and bone resorption [65,66]. RANKL is a transmembrane ligand synthesized by synovial cells and lymphocytes that induce osteoclast differentiation and maturation resulting in bone resorption [67]. On the other hand, the RANK/RANKL function is balanced by the OPG, which competes with RANKL for binding to RANK and thus negatively regulates osteoclast differentiation [68,69]. It is suggested that an imbalance between OPG and RANKL contributes to the hemophilic bone changes evidenced by decreased OPG levels and the strong expression of RANK and RANKL [70]. 

Oxidative stress is involved in the pathogenesis of bone loss in many disorders by favoring osteoclastogenesis, inhibiting the mineralization, and reducing osteoblast activity [71,72,73]. The expression of RANKL and OPG is sensitive to increased oxidative status, which induces RANKL up-regulation and OPG down-regulation through the activation of protein kinases of downstream signaling including mitogen activated protein kinases (ERK1/2, JNK) [74,75,76]. Moreover, oxidative stress blocks the activation of osteoblasts and induces osteocyte apoptosis, causing an imbalance in favor of osteoclastogenesis [77].

To date, limited experimental studies have targeted the direct antioxidant effects on bone remodeling. However, limited data suggest a correlation between reduced plasma antioxidant levels and osteoporosis [78,79,80]. Furthermore, antioxidants such as vitamin C, E, and N-acetylcysteine (NAC) might positively affect bone health in individuals with osteoporosis [81,82,83]. Together, these findings suggest a role of oxidative stress in cartilage/bone damage in hemophilic arthropathy, which may underlie the pathobiology of pain in hemophilia.

### 3.3. Inflammation

Iron and other blood components play a role in initiating the inflammatory process in hemarthropathy. This inflammatory process involves the synovial tissue, i.e., synovitis, and is characterized by recruitment of inflammatory cells, tissue hypertrophy, and neoangiogenesis [84]. Recent studies highlight the crucial role of inflammasome in the hemophilic arthropathy-related inflammatory process, which regulates the production of pro-inflammatory cytokines, including IL-1β. Moreover, IL-1α, IL-6, IL-1β, and TNF-α activate monocytes/macrophages [35]. The activation of these phagocytic cells may contribute to tissue damage in multiple ways. They trigger the release of proteases such as matrix metalloproteinases (MMPs), the production of inducible nitric oxide (NO), and tissue plasminogen activation [85,86]. These, in turn, play a role in activating other cells, including T cells, fibroblasts, and osteoclasts, through a variety of inflammatory mediators, resulting in articular cartilage and subchondral bone destruction [35]. 

Activated phagocytic cells contribute to oxidative stress in hemarthropathy. As a part of their defensive role, activated phagocytic cells undergo an oxidative burst, producing ROS that target and kill pathogens [87]. The phagocytic cells undergo the oxidative burst via the NADPH oxidase (NOX) system. This results in increased oxygen consumption and superoxide (O_2_^−^) production, which is converted to H_2_O_2_ either spontaneously or catalyzed by superoxide dismutase (SOD) [88]. SOD is present in three isoforms; of which the *SOD1* gene is often constitutively expressed and not as easily inducible as other SODs. At the same time, many inflammatory cytokines have been implicated in the induction of *SOD2,* including interferon-gamma (IFN-γ), TNF-α, IL-1β, IL-4, and IL-6, which are capable of rapidly modulating *SOD2* gene transcription [89]. NF-κB was identified as the most crucial transcriptional factor regulating cytokine-mediated induction of *SOD2* gene expression [90]. Moreover, the presence of excess iron contributes to a surge in hydroxide (OH^−^) and hydroxyl radical production via the Fenton reaction [84].

In addition to the oxidative burst role played by the phagocytic cells, neutrophils also contribute to oxidative stress. Remarkably, the neutrophil-associated enzyme myeloperoxidase can oxidize halides such as chloride (Cl^−^) and convert hydrogen peroxide into hypochlorous acid (HOCl). As a result, H_2_O_2_ can further interact with HOCl, producing singlet oxygen, another highly reactive and damaging radical [91]. 

### 3.4. Angiogensis

Angiogenesis is a hallmark feature of several arthritic conditions, including hemophilic arthropathy. Vascular endothelial growth factor (VEGF), a potent angiogenic factor, was elevated in patients with severe hemophilia both in circulation and synovial tissue [92,93]. Zetterberg et al. showed that angiogenesis is increased in advanced hemophilic joint disease with increased expression of VEGF and microvessel density [94]. This correlation between disease activity and high serum VEGF-A levels has also been shown in rheumatoid arthritis (RA) [37]. Interestingly, VEGF-A expression is induced essentially by hypoxia [35,92]. A study that involved collagen-induced arthritis (CIA) in mice demonstrated that tissue oxygenation is dysregulated in response to movement in arthritic mice compared to healthy non-arthritic mice [95]. Exposure to hypoxic conditions favors the increase of ROS, and despite the proliferation of new blood vessels, they are dysfunctional and fail to restore tissue-oxygen homeostasis [96].

NOX pathway is also active in nonphagocytic cells, including endothelial cells, vascular smooth muscle cells, fibroblasts, and chondrocytes [97,98,99]. It may be involved in the proliferation, differentiation, and migration of endothelial cells and thus contribute to the angiogenesis process [100,101,102], creating repetitive cycles of hypoxia and reoxygenation that favor chronic oxidative stress within the microenvironment of the affected arthritic joint [103,104]. NOX-1 silencing and antioxidants treatment were demonstrated to decrease angiogenic response of endothelial cells [105,106], suggesting a contribution of iron and oxidative stress in the promotion of dysfunctional angiogenesis. These findings implicate the active role of NOX–derived ROS in angiogenesis, presenting oxidative stress as a possible initiating factor in driving angiogenesis and thus a potential therapeutic target.

## 4. Oxidative Stress as a Contributor to Pain in Hemophilia

Recurrent joint bleedings result in chronic pain, which is more complicated than acute pain and adversely affects PWH. The underlying mechanism of pain in hemophilia remains to be elucidated [11,30,107,108,109,110]. Based on other chronic pain conditions with joint pain such as osteoarthritis (OA) or rheumatoid arthritis (RA), the possible mechanisms may include features of nociceptive and/or neuropathic pain [111,112]. Pain is currently categorized into nociceptive pain (often inflammatory) resulting from tissue damage; neuropathic pain involving injury to the somatosensory nervous system; and idiopathic pain, which has no identified cause [113,114,115]. These distinctions are important because of the specificity of analgesic therapy to be used. Correctly identifying when chronic pain can be classified as neuropathic is vital because common analgesics, such as non-steroidal anti-inflammatory drugs (NSAIDs), poorly control neuropathic pain [116]. Neuropathic pain is more likely to respond to nonstandard analgesia (including tricyclic antidepressants, 5-hydroxytryptamine–noradrenaline reuptake inhibitors, or gabapentinoids) than to NSAIDs. Nociception is the neural process of encoding noxious stimuli [115]. Oxidative stress contributes to the complex and profound changes that underlie clinical disorders associated with pain. In this section, we highlight the role of oxidative stress as an underlying mechanism for chronic pain in hemophilic arthropathy and a possible target for future therapeutic trials.

### 4.1. Nociceptive Pain

Oxidative stress refers to a condition where the production of oxidants exceeds the capacity for their neutralization, which causes damage to cell membrane, lipids, nucleic acids, and proteins [117]. For decades, the connection between oxidative stress status and nociception has been evident [118]. ROS and reactive nitrogen species (RNS) have been shown to contribute directly to the destructive and proliferative synovitis in persons with RA [119]. This pathobiology may have similarities to another inherited blood disorder, sickle cell disease (SCD), accompanied by chronic and acute pain in a microenvironment replete with cell-free heme, oxidative stress, inflammation, and pain [120]. Recent observations have also suggested that oxidative stress might be involved in bone pathogenesis, including osteoporosis, bone cancer development, diabetes-induced bone complications, and inflammatory joint diseases [121].

Marked inflammation and synovial hypertrophy noted in hemophilic arthropathy resemble the pathological mechanisms observed in RA, while the progressive degeneration of the hyaline cartilage mimics that seen in OA [84]. These processes may occur in parallel leading to degenerative arthritis that progresses until the joint is destroyed [36]. Joint inflammation earlier in life could be a key factor in the development and maintenance of chronic arthritic pain. Early joint inflammation ‘primes’ the joint by sensitizing the peripheral nerve fibers leading to acute pain [122]. This may occur through sensitization of primary afferents in the periphery as well as central sensitization of the spinal second order neurons leading to sustained chronic pain [123]. It is believed that both inflammatory and oxidative processes contribute to the pathogenesis of the acute nociceptive phase; however, oxidative stress accounts for the maintenance of the nociceptive process in the chronic phase [124].

The pathogenesis of hemarthropathy encompasses the recruitment of inflammatory cells resulting in the production of ROS (e.g., H_2_O_2_, superoxide (2O_2_), and peroxynitrite (ONOOH)) [125,126]. Oxidative stress degrades cellular membranes, nucleic acids, and extracellular components, leading to the accumulation of damaged proteins in the tissue [117]. Degradation products and cellular content containing oxidized molecules contribute to the exacerbation of synovial inflammation and exacerbation of pain [127]. Free radicals oxidize lipids such as linoleic acid (LA) or arachidonic acid (AA) into their active metabolites. Oxidized LA and AA activate the transient receptor potential vanilloid 1 (TRPV1), a multimodal receptor involved in the transduction of nociceptive and thermal stimuli [128]. Patwardhan et al. demonstrated that the injections of oxidized LA metabolites into rodent hind paw elicited spontaneous nocifensive behavior and thermal hyperalgesia via a TRPV1-dependent mechanism [129]. Recent studies have implicated that nerve growth factor (NGF) triggered the development of persistent thermal and mechanical hyperalgesia mediated by oxidative processes via the production of endogenous TRPV1 agonists and activation of the channel [130]. Moreover, the administration of the combined oxidative enzyme inhibitor/antioxidant significantly reduced thermal and mechanical nociception, denoting a positive role for oxidative processes in persistent nociception from NGF [131]. Both H_2_O_2_ and ONOO^−^ are involved in pain deriving from inflammation, mainly through the cyclooxygenase-2 (COX2)/prostaglandin E2 (PGE2) pathway [127]. PGE2 contributes to the generation of both inflammatory and neuropathic pain conditions, acting on four G protein-coupled receptor’s (GPCR) E prostanoid receptor subtypes 1, 2, 3, and 4 (EP1-4). PGE2 directly excites nociceptors and potentiates the sensitizing effects of other pain mediators such as ATP, bradykinin, and capsaicin [132]. This body of literature supports that oxidative stress is associated with and contributes to maintenance of chronic pain [118]. This contribution could be nociceptive (inflammatory) as well as neuropathic.

### 4.2. Neuropathic Pain

The poor correlation between the radiologic signs of arthritis and severity of pain and the dissociation between disease progression and pain in RA and OA drew attention to a potential risk of misunderstanding the underlying pain mechanisms in PWH [30]. Neuropathic pain may persist long after the initiation of nerve injury, and/or may arise spontaneously or be evoked by innocuous stimuli [133,134,135]. A recent study detected a positive neuropathic pain component when PWH completed pDq, a questionnaire that identifies neuropathic components in an individual´s pain profile [30]. Moreover, non-bony ankylosed knees from hemophilia patients with greater pain showed increased protein gene product (PGP) 9.5 positive sensory nerve fiber sprouting and NGF concentrations when compared to bony-ankylosed knee of end-stage hemophilic arthropathy with milder pain [136]. Impaired conditioned pain modulation response in PWH is suggestive of the involvement of the descending inhibitory pain pathway [137]. This observation comes in line with pain-related chronic conditions that share many of the underlying features of joint damage as hemophilic arthropathies such as OA and RA. Initial studies in these chronic arthritic conditions showed nociceptive and neuropathic pain features. Interestingly, sprouting of aberrant nerve fibers was detected in arthritic joints in experimental animal model where arthritis was induced by injecting complete Freund’s adjuvant (CFA), which contributed to pain-related behavior [138,139]. The increased density of unmyelinated nociceptive C-fibers expressing calcitonin gene-related peptide (CGRP) and sympathetic fibers into areas of inflammation may contribute to the persistence of pain when inflammation has subsided [139]. Arthritic human joints have shown similar CGRP+ nerve fiber sprouting and were associated with neovascularization following joint injury [122,140].

Growing evidence points to endoneural oxidative stress as a leading cause of nerve dysfunction and neuropathic pain [141]. Among neuropathic pain models, experimentally induced chronic constriction injury (CCI) of the sciatic nerve has been widely used to induce neuropathic pain in animals comparable to that observed in patients with neuropathic pain [142]. Studies have shown that oxidative stress mainly acts through mitochondrial dysfunction and the accumulation of lipid peroxidation products such as 4-hydroxynonenal (4-HNE) and malondialdehyde. The reduction of antioxidant peptide glutathione and increased expression of superoxide dismutase were identified as essential determinants of neuropathological and behavioral consequences of CCI-induced neuropathy [143].

The contribution of oxidative stress to neuropathic pain in the central and peripheral systems are multifactorial [144,145,146,147]. The nervous system is particularly vulnerable to oxidative stress due to its high levels of phospholipids and axonal mitochondrion and its relatively weak antioxidant defense [148]. It is possible for ROS to directly modulate neuroexcitability in central synapses by increasing glutamate release from primary afferent terminals and inhibiting GABAergic interneurons [146]. Superoxides have been shown to upregulate kinase signaling in hippocampal neurons [149,150]. The involvement of signal transduction pathways, particularly protein kinase C activation, is central in the development of peripheral and central sensitization of several pain etiologies [151,152,153,154,155]. It has also been shown that ROS may lead to the release of apoptotic factors from mitochondria, resulting in the degeneration of primary afferents. Activation of apoptosis contributes to the development of neuropathy [147]. Thus, the oxidative stress replete microenvironment in hemophilia is a potential contributor to neuropathic characteristics of pain and thus a promising treatable target.

## 5. Novel Antioxidant Therapies for Chronic Pain in Hemophilia

The Sirtuin (SIRT) family of proteins is amongst the most promising antioxidant agents [156]. The SIRTs are believed to play an essential role in the cellular response to various stresses, such as oxidative stress [157]. SIRT1 agonists modulate inflammation, oxidative stress, and mitochondrial dysfunction in a manner that can reduce chronic pain [158]. Several studies have shown that SIRT3 can enhance the ability of mitochondria to reduce ROS levels and protect against oxidative stress through regulating vital antioxidant enzymes, such as manganese superoxide dismutase (MnSOD) [159,160,161]. There is compelling evidence that polyphenols, including resveratrol, bergamot, hydroxytyrosol, and oleuropein, have the ability to activate SIRTs directly or indirectly [160,162,163,164]. The resveratrol compounds bind to SIRT1 at the N-terminal and increase SIRT1 activity [160], indicating its potential for treating chronic neuropathic pain [165]. Curcumin is another natural polyphenol that exhibits a neuroprotective role mediated by SIRT1 induction [166]. 

Another family of antioxidants are flavonoids that have been shown to improve and control diverse pain biomarkers in animal models of different neuropathic conditions, including diabetic- and chemotherapy induced-neuropathy [167]. Flavonoids are potent allosteric modulators of GABAergic receptors controlling their actions [168]. Essentially, flavonoids block oxidative stress, activate glial cells, and prevent mitochondrial dysfunction to reduce peripheral neuropathic pain [169]. 

Probucol is an anti-oxidative and lipid-lowering drug shown in preclinical and clinical studies to reduce serum low-density lipoprotein-cholesterol (LDL-C) [170]. In addition, probucol has been shown to counteract CCI-induced neuropathy via modulating NF-κB/NLRP3 signaling and augmenting transcription factor nuclear related factor (Nrf-2) activity [171]. Current evidence suggests that Nrf2 signaling contributes to antinociceptive activity by reducing inflammation, oxidative stress, and mitochondrial dysfunction [172].

In a recent study, vitamin D3 showed an antinociceptive effect in rats with CCI preventing the increase in lipid hydroperoxide, superoxide anion generation, and hydrogen peroxide (H2O2) levels in the spinal cord [173]. Thus, reduction in oxidative stress may be contributing to vitamin D-induced antinociception in CCI rats.

An unmet need is to decipher the mechanisms of oxidative stress underlying hemophilic arthropathy and pain to develop treatable targets. Several antioxidants described above are nutraceuticals and may not be toxic and/or interfere with current therapies for hemophilia. Thus, mechanism-based targeting of oxidative stress with novel antioxidants to prevent and/or treat hemophilic arthropathy and pain requires attention.

## 6. Conclusions

Increased bleeding, release of cell free heme, and subsequent cytokine elevation in joints may contribute to tissue damage and peripheral nerve fiber activation in hemophilia. Together, these phenomena may drive pain in hemophilia via oxidative stress as one of the major contributors to tissue damage and pain. Production and accumulation of ROS would promote immune cell activation and pro-inflammatory cytokine release, which may promote nociceptor activation leading to exacerbation of pain. It is likely that targeting mechanisms of oxidative stress in hemophilia with novel antioxidants may ameliorate pain and tissue damage.

## Data Availability

Data are available upon request.

## Abbreviations

PWH	Patients With Hemophilia
HRQoL	Health-Related Quality Of Life
pDq	Paindetect-Questionnaire
FLS	Fibroblast-Like Synoviocytes
IL	Interleukin
TNF-α	Tumor Necrosis Factor-Alpha
ROS	Reactive Oxygen Species
H_2_O_2_	Hydrogen Peroxide
OA	Osteoarthritis
SCD	Sickle Cell Disease
MMPs	Matrix Metalloproteinases
NO	Nitric Oxide
NOX	Nadph Oxidase
O_2_^−^	Superoxide
SOD	Superoxide Dismutase
Cl^−^	Chloride
HOCl	Hypochlorous Acid
VEGF	Vascular Endothelial Growth Factor
RA	Rheumatoid Arthritis
CIA	Collagen-Induced Arthritis
PAR-1	Protease-Activated Receptors
OPG	Osteoprotegerin
RANKL	RANK Ligand
NAC	N-Acetylcysteine
NSAIDs	Non-Steroidal Anti-Inflammatory Drugs
ONOOH	Peroxynitrite
LA	Linoleic Acid
AA	Arachidonic Acid
TRPV1	Transient Receptor Potential Vanilloid 1
OLAMs	Oxidized LA Metabolites
NGF	Nerve Growth Factor
COX2	Cyclooxygenase-2
PGE2	Prostaglandin E2
GPCR	G Protein-Coupled Receptors
EP1-4	E Prostanoid Receptor Subtypes 1, 2, 3 And 4
PGP 9.5	Protein Gene Product 9.5
CFA	Complete Freund’s Adjuvant
CGRP	Calcitonin Gene-Related Peptide
CCI	Chronic Constriction Injury
4-HNE	4-Hydroxynonenal
MDA	Malondialdehyde
